# Practical Histology in Vienna, and the Microscopical Study of Blood and Epithelium

**Published:** 1873-12

**Authors:** 


### BIBLIOGRAPHICAL.

*Practical Histology in Vienna, and The Microscopical Study of Blood and Epithelium.* Reprinted from the Philadelphia *Med. Times.* By James Tyson M. D. Lecturer on Microscopy and Urinary Chemistry in the University of Penn. etc. etc. Published by J. B. Lippincott & Co., Philadelphia, Pa.

The first part of this pamphlet relates to a recent brief experience of the author in the laboratory of Prof. Stricker, of Vienna, connected with the study of connective tissue. The second part contains researches into Blood and Epithelium by the aid of the microscope, and presents many interesting facts relating to their substances.

